# Integrative Transcriptomic and Metabolomic Analysis Reveal Mechanisms Underlying Differential Fecundity in Yangtze River Delta White Goat

**DOI:** 10.3390/ani16132034

**Published:** 2026-07-02

**Authors:** Jiahao Sun, Wenjun Tang, Rahmani Mohammad Malyar, Fangxiong Shi

**Affiliations:** 1 College of Animal Science and Technology, Nanjing Agricultural University, Nanjing 210095, China; 2022205014@stu.njau.edu.cn (J.S.); 2022105018@stu.njau.edu.cn (W.T.); rahmanimalyar@gmail.com (R.M.M.); 2 Lishui Institute of Agriculture and Forestry Sciences, Lishui 323000, China; 3 Veterinary Science Faculty, Nangarhar University, Jalalabad 2601, Afghanistan

**Keywords:** fecundity, goat, follicular, thyroid, uterine, metabolome, transcriptome

## Abstract

Differential fecundity in goats is a complex trait governed by coordinated molecular regulation across reproductive and endocrine tissues. In this study, we performed integrated metabolomic profiling of follicular fluid, serum, thyroid tissue, and uterine luminal fluid together with transcriptomic sequencing of follicular, thyroid, and uterine horn tissues from high-fecundity (HF) and low-fecundity (LF) Yangtze River Delta White goats. In addition, weighted gene co-expression network analysis (WGCNA) was conducted to elucidate the molecular mechanisms underlying differential litter size. High-fecundity goats exhibited enhanced follicular steroidogenesis, superior corpus luteum function, and more stable hypothalamic–pituitary–thyroid (HPT) axis activity, accompanied by increased uterine gland density and greater myometrial thickness. Metabolomic profiling identified 6640 metabolites displaying tissue-specific differential abundance patterns. Pathway enrichment analysis highlighted steroid hormone biosynthesis and energy metabolism in follicular fluid, PPAR signaling and tyrosine metabolism in thyroid tissue, and glycerophospholipid and one-carbon metabolism in uterine luminal fluid as major pathways associated with fecundity. Transcriptomic analysis identified 1596 differentially expressed genes (DEGs), including 20 genes shared across all examined tissues, constituting a systemic molecular signature associated with fecundity. WGCNA further revealed three functional tissue axes associated with follicular development (*ELOVL4*, *INHA*, *NR5A2*), thyroid endocrine regulation (*GRHL2*, *NAPRT*), and uterine receptivity (*RSPO1*, *AGTR2*, *PTGER3*). Low-fecundity-associated modules were predominantly concentrated in follicular and thyroid tissues, whereas the high-fecundity-specific module was mainly enriched in uterine horn. These findings provide a multi-tissue molecular framework underlying differential fecundity in goats and identify candidate hub genes and metabolites that may serve as candidate biomarkers for fecundity assessment and selective breeding programs.

## Simple Summary

Litter size in goats varies substantially among individuals; however, the molecular mechanisms underlying these differences remain insufficiently understood. In this study, high-fecundity and low-fecundity Yangtze River Delta White goats were compared through integrated transcriptomic and metabolomic analyses of follicular, thyroid, and uterine tissues and fluids. High-fecundity goats exhibited enhanced ovarian steroidogenesis, more stable hypothalamic–pituitary–thyroid (HPT) axis activity, and improved uterine conditions conducive to embryo implantation. Metabolomic analysis identified steroid hormone biosynthesis and energy metabolism as major pathways associated with follicular development, while transcriptomic analyses revealed 20 genes consistently altered across all examined tissues, forming a systemic molecular signature associated with fecundity. Network analysis further identified key candidate genes involved in follicular maturation, thyroid endocrine regulation, and uterine receptivity. Collectively, these findings suggest that reproductive performance in goats is associated with coordinately regulated by the ovary, thyroid gland, and uterus through interconnected molecular pathways. The identified candidate genes and metabolites may serve as candidate biomarkers for genetic selection and breeding programs aimed at improving litter size in goats.

## 1. Introduction

Fecundity is one of the most economically important reproductive traits in small ruminant production because litter size directly influences the production efficiency of meat, fiber, and dairy products per breeding female [1]. Litter size in goats is shaped by factors including genetic, physiology, breed-related, and environmental factors [2]. The Yangtze River Delta White (YRDW) goat is a Chinese indigenous breed listed on the National List of Livestock and Poultry Genetic Resources. It is characterized by stable genetic performance and good adaptability, making it an ideal model for studying reproductive physiology and fecundity regulation [3,4]. However, the molecular and physiological mechanisms governing its fecundity variation are not fully understood. Therefore, uncovering the genetic architecture underlying differential fecundity in the YRDW breed remains a priority in livestock genetics and reproductive biology.

Reproductive performance in mammals depends on the coordinated interaction of multiple organs and endocrine systems, particularly the ovaries, uterus and thyroid glands. The follicular microenvironment, especially follicular fluid, plays a central role in regulating oocyte maturation, ovulation, and early embryonic development [5]. Follicular fluid contains protein, steroid hormones, cytokines, reactive oxygen species (ROS), antioxidants, growth factors, and numerous metabolites that collectively influence follicular development and oocyte competence [6]. Similarly, the uterus provides the structural and physiological environment necessary for embryo implantation and pregnancy maintenance, while uterine gland development and luminal fluid composition are essential determinants of endometrial receptivity [7,8]. Increasing evidence indicates that differentially expressed genes and metabolites in ovarian and uterine tissues are involved in steroid biosynthesis, energy metabolism, and signal transduction pathways that regulate goat follicular development and sexual maturation [9,10]. As a central endocrine organ, the thyroid gland modulates systemic metabolism and reproductive activity through the hypothalamic–pituitary–thyroid (HPT) axis, and thyroid dysfunction is closely linked to impaired folliculogenesis, abnormal estrous cycles, and reduced pregnancy rates [11,12]. Disruption of the HPT axis interferes with hypothalamic GnRH pulsatility and ovarian follicular development, ultimately compromising reproductive competence [13].

Transcriptomics and metabolomics have proven useful as complementary tools for elucidating the molecular underpinnings of complex reproductive traits. Transcriptomic analysis identified differentially expressed genes in the ovary and uterus, such as *FOXC1*, *FOSB*, and *FGL2*, providing potential molecular marker for litter size traits [14,15]. Single-cell RNA sequencing demonstrated that paracrine crosstalk between granulosa cells and macrophages via *IL1RAP*, *COL4A1/2*, and *EREG* plays a critical role in regulating immune responses and oocyte maturation [16]. Transcriptomic and proteomic analyses revealed a marked upregulation of the relaxin signaling pathway during the follicular phase, whereas the terpenoid backbone biosynthesis pathway exhibited heightened activity in the luteal phase [17]. Integrated metabolomic and transcriptomic analyses revealed that oocyte maturation entails key regulators, including *SCARB1*, *CYP11A1*, *3BHSD*, progesterone, estradiol, and L-phenylalanine [9], and metabolic alterations, such as active serine metabolism, enhanced tryptophan utilization, and accumulation of purine nucleotide [18]. Serum metabolomics distinguished predictive biomarkers of pregnancy and litter size in sheep including specific lipid and amino acid species as early as 50 days post-mating [19]. However, most current studies have examined either transcriptomics or metabolomics in isolation and have focused on a single tissue type, providing only a partial view of the systemic molecular changes underlying differential fecundity. An integrated multi-omics analysis across multiple tissues and biofluids is necessary to achieve a more comprehensive understanding of the regulatory mechanisms underlying high fecundity.

In this study, we integrate transcriptomic and metabolomic data to characterize the molecular differences between high-fecundity (HF) and low-fecundity (LF) YRD does. Transcriptome sequencing was performed on ovarian follicles, uterine horn tissues, and thyroid glands, while non-targeted metabolomics was applied to matched follicular fluid, uterine fluid, serum, and thyroid tissue samples. Using network-based integration, we aimed to identify key genes, metabolites, and shared pathways associated with fecundity, and to establish regulatory networks across reproductive and endocrine tissues. Our findings provide new mechanistic insights into the molecular regulation of fecundity in the YRDW goat and offer potential molecular targets for genetic improvement of reproductive performance in indigenous goat breeds.

## 2. Materials and Methods

### 2.1. Animal Experiments and Sample Collection

Experimental goats were obtained from the Zhejiang Yangtze River Delta White Goat Conservation Farm of Lishui Guge Ecological Company (Lishui, China). Multiparous non-pregnant goats in clinically healthy condition, without genetic abnormalities, and maintained under identical feeding and management conditions were selected for the experiment. Goats that produced fewer than two kids in at least two consecutive pregnancies were assigned to the low-fecundity group (LF, *n* = 11), whereas goats that produced more than two kids in at least two consecutive pregnancies were assigned to the high-fecundity group (HF, *n* = 8). All goats were aged 2–4 years, had a parity number > 2, and had body condition scores of 2.5–3.5. All animals were maintained under the same environmental and nutritional conditions. Estrus synchronization was performed to standardize the reproductive cycle among animals. Blood samples were collected between 7:00 and 9:00 a.m. on days 0, 4, 10 and 16 of the second estrus cycle to minimize circadian and hormonal variation. Three goats from each group were randomly selected and humanely slaughtered during the second estrus cycle. Medium-sized follicles, thyroid tissues, and the upper one-third of the uterine horn were immediately snap-frozen in liquid nitrogen and stored at −80 °C for transcriptomic analysis. Serum, follicular fluid, uterine luminal fluid, and thyroid tissue samples were used for untargeted metabolome analysis.

### 2.2. Endometrial Receptivity Analysis

Uterine morphological analysis was performed as follows: one uterine horn was incised to count the number of endometrial caruncles, and its lumen was flushed with 200 µL of physiological saline to collect uterine luminal fluid for pH measurement. For histological quantification, tissue sections were examined under a light microscope at 20× magnification. Microvessels were defined as discrete brown endothelial cell clusters distinct from the stroma, and ductal gland invaginations (DGI) were identified as individual glandular epithelial invaginations. In each section, five randomly selected fields of view were used to quantify uterine gland density, microvessel number, and DGI count. Additionally, endometrial thickness and myometrial thickness were measured at ten randomly chosen locations per section.

### 2.3. Metabolomics Analysis

Ultra-high performance liquid chromatography–tandem mass spectrometry (UPLC-MS/MS, Thermo Scientific, Waltham, MA, USA) was used to detect metabolites in pretreated samples. Peak extraction, alignment, and retention time correction were performed using the XCMS program. Peaks with a missing rate > 50% in each group were filtered out, blank values were imputed by K-nearest neighbor (KNN, version 1.56.0) algorithm, and peak areas were corrected using the support vector regression (SVR, version XXX) method. The corrected and filtered peaks were identified and annotated by searching the laboratory self-built database, integrating public databases (KEGG, HMDB, METLIN, and CAS), prediction databases, and the metDNA method. OPLS-DA models (version 1.0.1) were validated by R^2^Y, Q^2^, and permutation testing (*n* = 200) to guard against overfitting. FDR was calculated by the Benjamini–Hochberg method for all metabolites and was provided in the Supplementary Tables S3–S6. Differential metabolites (DEMs) were defined by VIP (VIP > 1, from OPLS-DA), *p*-value (*p* < 0.05, Student’s *t* test), |log_2_(fold change)| ≥ 1 for subsequent analysis.

### 2.4. Transcriptome Sequencing and Analysis

Three goats from each group were randomly selected as biological replicates for transcriptomic analysis. Total RNA was extracted from goats using the Trizol reagent (Tiangen, Beijing, China). The concentration and integrity of RNA were detected by an Agilent 5400 analyzer (Agilent Technologies, Palo Alto, CA, USA), and agarose gel electrophoresis was used to assess RNA purity. Qualified RNA samples were sent to Novogene (Beijing, China) for library construction and transcriptome sequencing. Raw sequencing reads were first quality-controlled and preprocessed. Adapter sequences and low-quality bases were trimmed using fastp (version 0.23.1). The clean reads were then aligned to the *Capra hircus* reference genome (NCBI ARS1.2) using HISAT2 (version 2.0.5). Gene expression levels were quantified as fragments per kilobase of transcript per million mapped fragments (FPKM). Differential expression analysis was performed with the edgeR (version 3.22.5) package with the raw count, and genes meeting the thresholds of |log_2_(fold change)| ≥ 1 and *p*-value < 0.05 were defined as differentially expressed genes (DEGs). The statistics for all DEGs are provided in Supplementary Tables S12–S14. Functional enrichment analysis of the DEGs was conducted for Gene Ontology (GO) terms and Kyoto Encyclopedia of Genes and Genomes (KEGG) pathways using the ClusterProfiler (version 3.8.1) R package.

### 2.5. Quantitative Real-Time PCR Validation

Primers were designed with Primer-BLAST (https://www.ncbi.nlm.nih.gov/tools/primer-blast/, accessed on 28 June 2026) and were listed in Table S1. qPCR assays were conducted on a QuantStudio 7 system (ABI, Waltham, MA, USA) with SYBR Green detection. The reaction mix comprised the following components in a final volume of 10 μL: 5 μL of 2× SYBR Green master mix (TRAN, Beijing, China), 0.2 μL of forward primer (20 pmol/μL), 0.2 μL of reverse primer (20 pmol/μL), 1 μL of cDNA, and 3.6 μL of nuclease-free water. The cycling conditions were: 95 °C for 2 min; 40 cycles of 95 °C for 10 s and 60 °C for 30 s. Each sample was run in three technical replicates. Relative expression was determined using the 2^−ΔΔCT^ method.

### 2.6. Construction of the Gene Co-Expression Network

Weighted gene co-expression network analysis (WGCNA) was performed separately on the DEG and DEM datasets using the R package WGCNA (version 1.73). The soft threshold power was selected to achieve a scale-free topology fit. For the analysis of genes, the soft threshold power was set to 18 (R^2^ > 0.6, Figure S2), the minModuleSize was set to 50, and mergeCutHeight was set to 0.25 for tissue- or stage-specific module detection. For the analysis of metabolites, the soft threshold power was set to 17 (R^2^ > 0.8, Figure S3), the minModuleSize was set to 15, and mergeCutHeight was set to 0.25 for tissue- or stage-specific module detection. Subsequently, the module significance (GS) and module membership (MM) of genes within the module were calculated and sorted in descending order, defining hub genes as those with a |MM| > 0.8. Cytoscape software (version 3.7.2) was used for network visualization.

### 2.7. Statistical Analysis

Statistical analysis was performed with SPSS 26.0, and graphs were generated using GraphPad Prism 10. Data are expressed as mean ± SEM. Inter-group differences were assessed by either the Mann–Whitney U test or Student’s *t*-test, based on normality distribution. Statistical significance was set at *p* < 0.05.

## 3. Results

### 3.1. Reproductive Phenotypic Differences Between Goats with Divergent Fecundity

**Figure 1:**
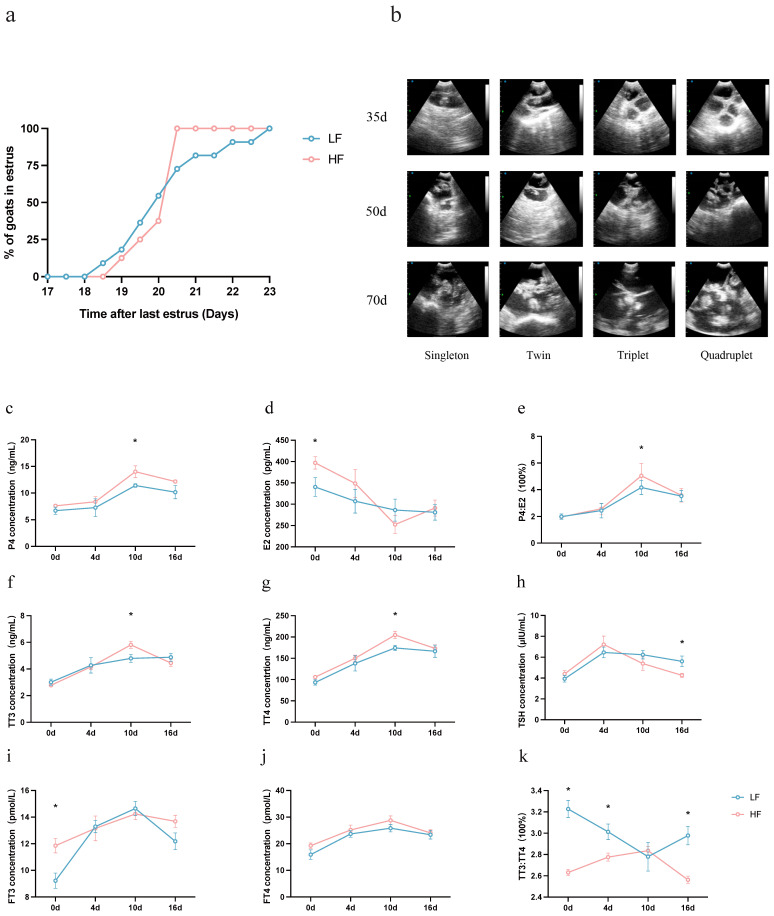
Reproductive performance and serum hormone levels in goats with different fecundity. (**a**) Cumulative estrus percentage in goats with different fecundity. (**b**) Representative USG images. (**c**–**k**) Comparison of serum hormone levels in goats with different fecundity during estrus cycle. Data are presented as mean ± standard error of the mean (SEM). * indicates *p* < 0.05.

A comparison of reproductive traits between the LF and HF groups is summarized in Table 1. No significant differences in estrus characteristics were observed between the LF and HF groups. During early and mid-gestation, the number of gestational sacs was significantly higher in the HF group (*p* < 0.05). The gestation period of the LF group was significantly shorter than that of the HF group (144.71 ± 2.07 d vs. 150.25 ± 1.25 d, *p* = 0.035). Repeated ultrasonographic monitoring during early gestation (35, 50, and 70 d; Figure 1b) consistently confirmed that the number of gestational sacs in the LF group was significantly lower than in the HF group (*p* < 0.05), which is consistent with the classification criterion based on cumulative litter records across consecutive parities. Actual litter size and total litter birth weight were significantly lower in the LF group than in the HF group (1.67 ± 0.24 vs. 3.00 ± 0.24; 3.42 ± 0.36 kg vs. 4.87 ± 0.30 kg; *p* < 0.01), whereas individual birth weight was significantly higher in the LF group (2.16 ± 0.14 kg vs. 1.67 ± 0.12 kg, *p* < 0.05), reflecting the classical trade-off between litter size and individual birth weight. However, at one month of age and at weaning, no significant differences in either litter weight or individual body weight were detected between the two groups (*p* > 0.05), indicating that the initial birth-weight disadvantage of HF offspring can be compensated by accelerated postnatal growth.

Serum hormone profiles across the estrous cycle (days 0, 4, 10, and 16; Figure 1c–k) revealed the following patterns. During the follicular phase (day 0), estradiol (E2) and free triiodothyronine (FT3) levels were significantly higher in the HF group (*p* < 0.05; Figure 1d,i). During the mid-luteal phase (day 10), progesterone (P4), total triiodothyronine (TT3), total thyroxine (TT4), and the P4/E2 ratio were all significantly elevated in the HF group (*p* < 0.05; Figure 1c,e–g). During the late-luteal phase (day 16), thyroid-stimulating hormone (TSH) was significantly higher in the LF group (*p* < 0.05; Figure 1h). The P4/E2 ratio, used here as an indicator of corpus luteum function, differed significantly between groups only at the mid-luteal phase (day 10; *p* < 0.05; Figure 1e). Because sex steroids such as E2 can interfere with thyroid hormone synthesis and metabolism through receptor-mediated signaling [20], the TT3/TT4 ratio was adopted as a corrected index of thyroid hormone conversion and metabolic homeostasis. The TT3/TT4 ratio was significantly higher in the LF group at days 0, 4, and 16 (*p* < 0.05; Figure 1k). Combined with the stage-specific elevation of TSH in the LF group during the late-luteal phase, these findings indicate aberrant negative-feedback regulation of the HPT axis in LF goats.

### 3.2. Uterine Morphological Analysis

**Figure 2:**
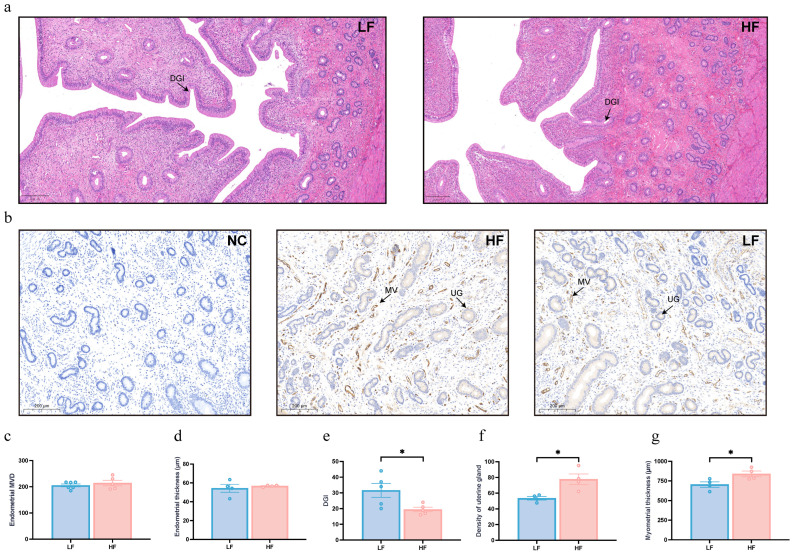
Uterine morphology in goat with different fecundity. (**a**) HE staining of uterine tissue. HF, high fecundity; LF, low fecundity; DGI, endometrial ductal gland invaginations; Bar = 200 µm. (**b**) Immunohistochemical staining of endometrium tissues. NC, negative control; HF, high fecundity; LF, low fecundity; MV, micro vessel; UG, uterine glands; Bar = 200 µm. (**c**) Analysis of endometrial microvascular density (MVD). (**d**) Analysis of endometrial thickness. (**e**) The number of DGI. (**f**) Analysis of density of uterine gland. (**g**) Analysis of endometrial myometrium thickness. Data are presented as mean ± standard error of the mean (SEM). * indicates *p* < 0.05.

Uterine tissue morphology, glandular architecture, and microvascular characteristics were further examined in goats with divergent fecundity (Figure 2). Hematoxylin and eosin (HE) staining revealed that luminal epithelial cells were densely arranged in both groups, with prominent sub-nuclear vacuolation, indicating active uterine secretory function at estrus in both groups. Qualitative morphological examination showed that the HF group had more proliferative uterine glands and fewer endometrial ductal gland invaginations (DGI) (Figure 2a). Quantitative analysis confirmed that the number of DGI was significantly higher in the LF group (*p* < 0.05; Figure 2e), whereas uterine gland density and myometrial thickness were both significantly lower than in the HF group (*p* < 0.05; Figure 2f,g). No significant differences in endometrial microvascular density (MVD) or endometrial thickness were observed between the two groups (*p* > 0.05; Figure 2c,d), indicating that basic structural integrity, microvascular supply, and luminal pH of the uterus were comparable between groups.

### 3.3. Differential Metabolomic Profiling Between Goats with Divergent Fecundity

**Figure 3:**
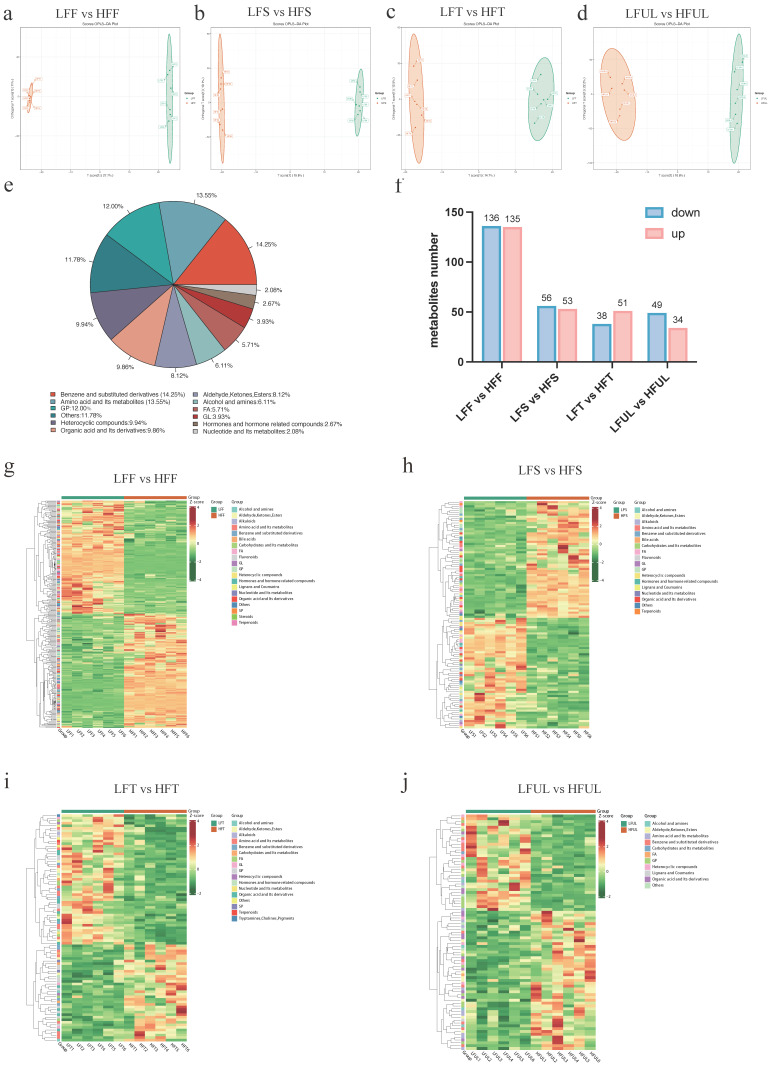
Metabolite profiles between the follicular fluid (F), Serum (S), thyroid (T) and Uterine fluid (UL). (**a**–**d**) OPLS-DA score plot. (**e**) Pie chart depicting the categories of metabolites. (**f**) The number of DEMs identified in different tissues. (**g**–**j**) Cluster analysis of DEMs identified in different tissues.

Non-targeted metabolomics by LC-MS was performed to investigate differences in metabolite composition in follicular fluid (F), serum (S), thyroid tissue (T), and uterine luminal fluid (UL) between HF and LF goats. Orthogonal partial least-squares discriminant analysis (OPLS-DA) revealed clear separation between groups in all four sample types (Figure 3a–d), confirming substantial metabolite differences between HF and LF groups. Permutation testing confirmed no overfitting, with R^2^Y values close to 1 and Q^2^ intercepts with the *y*-axis were negative for all models (Figure S1).

A total of 6640 metabolites were identified, predominantly comprising benzene and its derivatives (14.25%), amino acids and their metabolites (13.55%), glycerophospholipids (12.00%), organic acids and their metabolites (9.86%), and hormones and related substances (2.67%) (Figure 3e; Table S2). These results indicate that fecundity-associated metabolic differences span energy metabolism, amino acid metabolism, and lipid metabolism. All metabolites were annotated against public databases. Differentially Expressed Metabolites (DEMs) were filtered using the criteria VIP > 1, *p* < 0.05, and |log_2_(fold change)| ≥ 1 (Figure 3f).

The number of down-regulated DEMs was slightly lower than that of up-regulated DEMs in LF vs. HF comparisons. The follicular fluid group (F) yielded the largest total number of DEMs (271; 136 up-regulated and 135 down-regulated; Table S3), followed by serum (S; 109 DEMs: 53 up-regulated and 56 down-regulated; Table S4) and thyroid (T; 89 DEMs: 51 up-regulated and 38 down-regulated; Table S5). The uterine luminal fluid group (UL) had the fewest DEMs (83; 34 up-regulated and 49 down-regulated; Table S6).

Differential metabolite abundance profiles showed clear tissue-specific clustering (Figure 3g–j). The F group exhibited the highest DEM abundance, while the UL group exhibited the lowest. Primary metabolites such as alcohols, amines, aldehydes, ketones, and esters were differentially represented across all four tissue types. Reproductive biofluids (F and UL) were enriched in energy and nutritional metabolites; thyroid tissue (T) was dominated by endocrine hormone metabolites; and serum (S) displayed broad-spectrum metabolic differences, collectively reflecting tissue-specific metabolic regulation associated with fecundity.

### 3.4. Functional Enrichment Analysis of Differentially Expressed Metabolites

**Figure 4:**
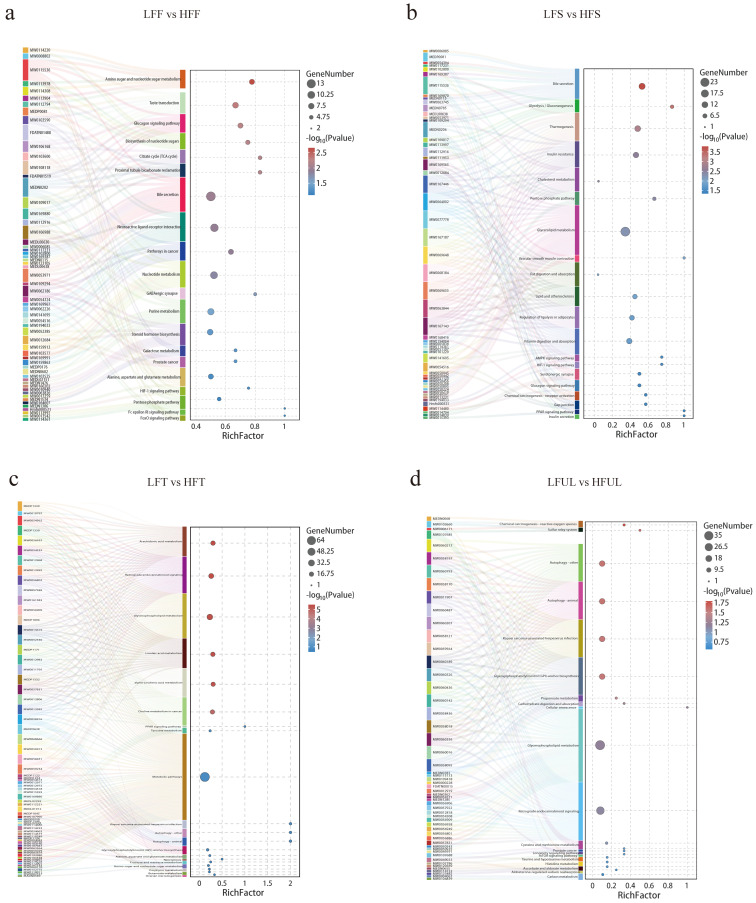
Functional enrichment analysis of DEMs in goats with different fecundity. (**a**) Functional enrichment in follicular fluid. (**b**) Functional enrichment in serum. (**c**) Functional enrichment in thyroid. (**d**) Functional enrichment in uterine fluid.

The top 20 enriched KEGG pathways were selected for pathway enrichment analysis. In the LFF vs. HFF comparison, pathways were primarily related to energy metabolism, reproductive hormone biosynthesis, signal transduction, and carbohydrate metabolism, including the TCA cycle, steroid hormone biosynthesis, neuroactive ligand–receptor interaction, and amino sugar and nucleotide sugar metabolism (Figure 4a). In the LFS vs. HFS comparison, enriched pathways were dominated by systemic basal metabolism, lipid metabolism, and energy regulation, encompassing glycolysis/gluconeogenesis, glycerophospholipid metabolism, cholesterol metabolism, the pentose phosphate pathway, and thermogenesis (Figure 4b). The HFT vs. LFT comparison showed the most pronounced pathway perturbations; combined with annotation from Sankey diagrams, the predominant enriched pathways were in lipid metabolism, nuclear receptor signaling, and amino acid metabolism, including α-linolenic acid metabolism, the PPAR signaling pathway, and tyrosine metabolism—representing the three core pathway categories of thyroid metabolic regulation (Figure 4c). The LFUL vs. HFUL comparison was primarily enriched in lipid metabolism, amino acid metabolism, cellular autophagy, and oxidative stress-related pathways, including glycerophospholipid metabolism, histidine metabolism, and cysteine and methionine metabolism (Figure 4d). In summary, glycolysis/gluconeogenesis, steroid hormone biosynthesis, neuroactive ligand–receptor interaction, the PPAR signaling pathway, tyrosine metabolism, and glycerophospholipid metabolism emerged as the core metabolic pathways regulating fecundity in does.

### 3.5. Differential Transcriptomic Profiling Between Goats with Divergent Fecundity

**Figure 5:**
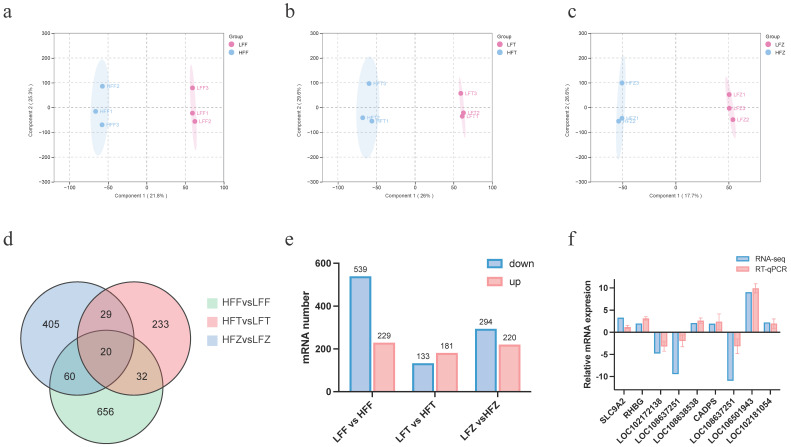
Transcriptomic profiles of the follicles (F), thyroid (T) and uterine horn (Z). (**a**–**c**) OPLS-DA score plots. (**d**) Pairwise Venn diagrams of DEGs. (**e**) The number of DEGs. (**f**) RT-qPCR validation of RNA-seq. data are expressed as mean ± SEM.

OPLS-DA based on FPKM values showed high intra-group consistency and clear inter-group separation (Figure 5a–c). DEGs were identified using the criteria *p* < 0.05 and |log_2_(fold change)| ≥ 1, yielding a total of 1596 DEGs (Figure 5d,e). In the LFF vs. HFF comparison, 768 DEGs were identified (229 up-regulated and 539 down-regulated). In the LFT vs. HFT comparison, 314 DEGs were identified (181 up-regulated and 133 down-regulated). In the HFZ vs. LFZ comparison, 514 DEGs were identified (220 up-regulated and 294 down-regulated). Notably, 20 DEGs were shared across all three tissue comparisons and may represent core cross-tissue regulatory hub genes for fecundity differences (Supplementary Table S12). Three DEGs from each comparison group were randomly selected from genes satisfying |log_2_FC| ≥ 1 for qRT-PCR validation. All validated genes showed expression trends consistent with RNA-seq results (Figure 5f), confirming the reliability of the sequencing data for subsequent functional analyses.

### 3.6. Functional Enrichment Analysis of Differentially Expressed Genes

**Figure 6:**
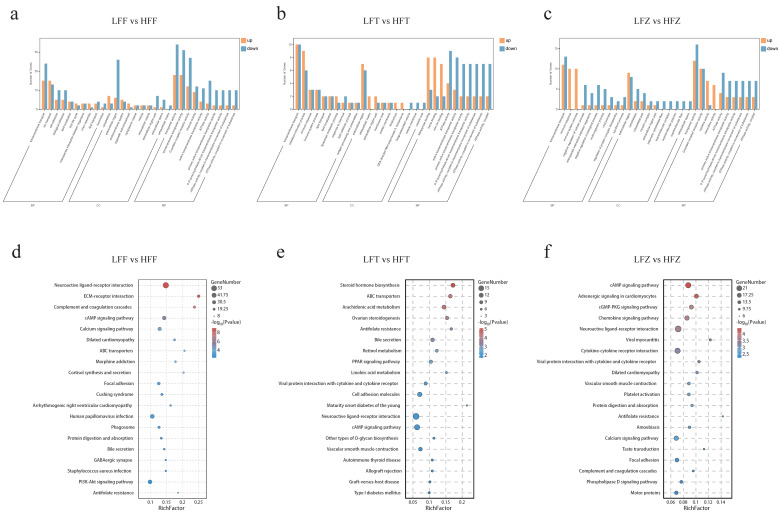
KEGG and GO enrichment analysis of DEGs in goats with different fecundity. (**a**) GO enrichment in follicles. (**b**) GO enrichment in thyroid. (**c**) GO enrichment in uterine horn. (**d**) KEGG enrichment in follicles. (**e**) KEGG enrichment in thyroid. (**f**) KEGG enrichment in uterine horn.

The top 10 most significantly enriched Gene Ontology (GO) terms in each category—biological process (BP), cellular component (CC), and molecular function (MF)—were annotated (Figure 6a–c). In the LFF vs. HFF comparison, the GO terms with the greatest numbers of enriched genes included transporter activity, transmembrane transporter activity, G protein-coupled receptor (GPCR) activity, transmembrane transport, ion transport, and extracellular region; the majority of enriched genes were down-regulated (Figure 6a). In the LFT vs. HFT comparison, up-regulated genes were mainly enriched in transmembrane transport, oxidation–reduction processes, and molecular functions such as iron ion binding, heme binding, and tetrapyrrole binding, whereas down-regulated genes were primarily associated with ATPase activity and transmembrane transporter activity (Figure 6b). In the LFZ vs. HFZ comparison, DEGs were predominantly enriched in biological processes such as transmembrane transport and immune response, and in molecular functions including transporter activity, GPCR activity, and ATPase activity, with enrichment patterns similar to those observed in the follicular group (Figure 6c).

For KEGG pathway analysis, the top 20 most significantly enriched pathways were annotated for each comparison (Figure 6d–f). In the LFF vs. HFF comparison, DEGs were mainly enriched in signal transduction pathways (PI3K-Akt, calcium signaling, cAMP, neuroactive ligand–receptor interaction), cell adhesion pathways (ECM–receptor interaction, focal adhesion), and immune-related pathways (complement and coagulation cascades) (Figure 6d). In the LFT vs. HFT comparison, enriched pathways spanned immune-related pathways (autoimmune thyroid disease, allograft rejection, graft-versus-host disease), reproductive hormone biosynthesis pathways (ovarian steroidogenesis, steroid hormone biosynthesis), signal transduction pathways (cAMP, neuroactive ligand–receptor interaction), and metabolic pathways (PPAR signaling, retinol metabolism, arachidonic acid metabolism) (Figure 6e). In the LFZ vs. HFZ comparison, DEGs were primarily enriched in signal transduction pathways (cAMP, calcium, cytokine–cytokine receptor interaction, neuroactive ligand–receptor interaction), immune and cardiovascular pathways (cGMP-PKG, complement and coagulation cascades, chemokine signaling, platelet activation), and cell adhesion pathways (focal adhesion) (Figure 6f).

Integrated GO and KEGG enrichment results reveal tissue-specific transcriptional regulatory programs associated with differential fecundity: Follicles tissue modulates follicular development through signal transduction, cell adhesion, and substance transport pathways; the thyroid maintains endocrine homeostasis via immune balance, steroid hormone biosynthesis, and metabolic pathways; and the uterine horn constructs uterine receptivity through signal transduction, immune, and cell adhesion pathways. These three tissues collectively contribute to fecundity differences through a coordinated network of signaling, immune regulation, metabolism, and material transport, providing a core mechanistic foundation for subsequent analyses.

### 3.7. WGCNA Co-Expression Module Identification and Pathway Enrichment

We performed weighted gene co-expression network analysis (WGCNA) to delineate tissue-specific metabolite-gene interactions. The analysis integrated metabolomic (F, T, UL) and transcriptomic (F, T, Z) data, comprising 6640 metabolites and 20,627 transcripts, to identify key co-expression modules and their hub genes/metabolites.

**Figure 7:**
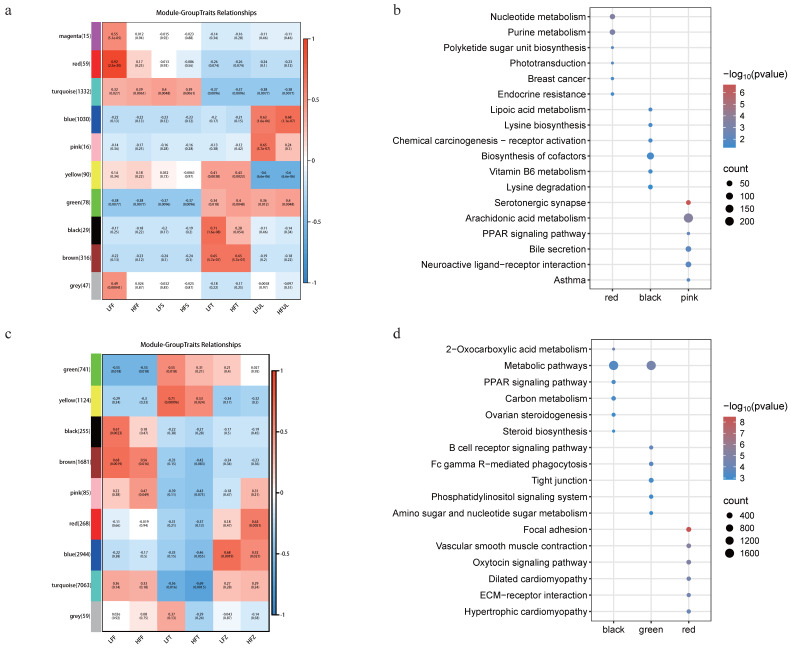
WGCNA and KEGG analysis of identified metabolites and transcriptomic genes. (**a**) Module–trait association heatmap of gene co-expression modules (**b**) KEGG pathway enrichment analysis for core metabolite modules (red, black, pink). (**c**) Module–trait association heatmap of metabolite co-expression modules (**d**) KEGG pathway enrichment analysis for core gene modules (black, green, red).

Ten modules were identified in the metabolite co-expression network, excluding the gray module (Figure 7a). The red module contained 59 metabolites significantly positively correlated with the LFF group (r = 0.92, *p* < 0.05); the black module contained 29 metabolites positively correlated with the LFT group (r = 0.71, *p* < 0.05); and the pink module contained 16 metabolites positively correlated with the LFUL group (r = 0.65, *p* < 0.05). KEGG pathway enrichment analysis of these three tissue-specific low-fecundity-associated modules revealed that they were jointly enriched in key pathways related to fecundity regulation, including neuroactive ligand–receptor interaction, nucleotide metabolism, the PPAR signaling pathway, and arachidonic acid metabolism (Figure 7b). These pathways are central to reproductive hormone signal transduction, lipid metabolic homeostasis, and endocrine regulation, further elucidating the molecular mechanisms by which metabolite modules participate in reproductive function across different tissues.

Gene co-expression network analysis classified all identified genes into nine modules (Figure 7c). The black module contained 255 genes positively correlated with the LFF group (r = 0.67, *p* < 0.05); the green module contained 741 genes positively correlated with the LFT group (r = 0.71, *p* < 0.05); and the red module was positively correlated with the HFZ group (r = 0.63, *p* < 0.05). KEGG pathway enrichment of these fecundity- and tissue-associated gene modules revealed predominant enrichment in ECM–receptor interaction, focal adhesion, cAMP signaling, steroid biosynthesis, oxytocin signaling, and amino sugar and nucleotide sugar metabolism (Figure 7d). These pathways are highly overlapping with the core pathways enriched in the metabolite modules, forming a metabolite–gene pathway regulatory co-network that jointly mediates key reproductive processes including follicular development, thyroid endocrine homeostasis, and uterine receptivity.

### 3.8. Identification of Key Hub Metabolites and Genes

**Figure 8:**
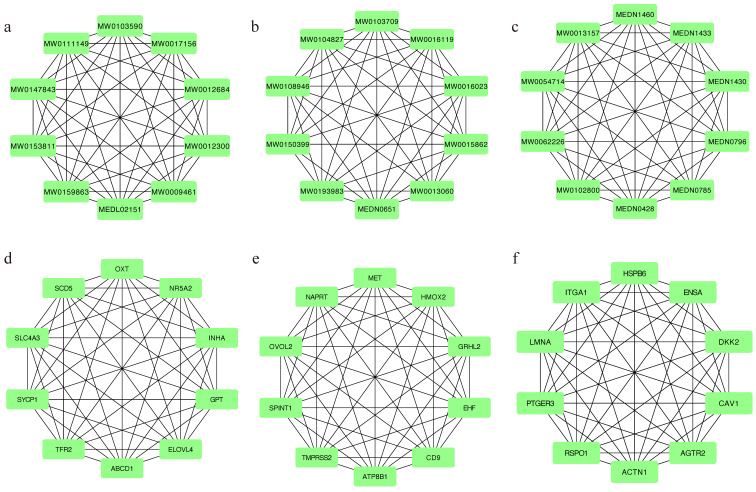
Co-expression network plots of hub metabolites (TOP10) and hub genes (TOP10). (**a**) red module (positively correlated with LFF). (**b**) black module (positively correlated with LFT). (**c**) pink module (positively correlated with LFUL). (**d**) black module (positively correlated with LFF). (**e**) green module (positively correlated with LFT). (**f**) red module (positively correlated with HFZ), sorted in descending order of weight value within the module.

Hub metabolites and hub genes met the criterion of module membership (|MM| > 0.8) in each co-expression module. Each module contributed its top 10 nodes by |MM| value to the hub list (Figure 8; Tables S16 and S17).

Metabolite co-expression network. Hub metabolites in the red module were predominantly free fatty acids, nucleotides and their metabolites, and hormone-related substances, including 13,16,19-docosatrienoic acid, hypoxanthine, and estradiol (Figure 8a). Hub metabolites in the black module were primarily organic acids and their derivatives, nucleotides and their metabolites, and carbohydrates, encompassing azelaic acid, uridine-5′-monophosphate, and polymannose (Figure 8b). Hub metabolites in the pink module were characterized by hormone-related substances, oxidized lipids, and nucleotides and their metabolites, including prostaglandin D2, LTB4, and N6-succinyl adenosine (Figure 8c).

Gene co-expression network. Hub genes in the black module were centered on follicular development, energy metabolism, and reproductive hormone regulation, including *ELOVL4*, *SCD5*, *INHA*, *OXT*, *GPT*, *TFR2*, *SLC4A3*, *NR5A2*, *ABCD1,* and *SYCP1* (Figure 8d), collectively regulating follicular maturation through pathways governing follicular cell proliferation and differentiation, energy metabolism, and hormone signal transduction. Hub genes in the green module focused on thyroid barrier maintenance, endocrine metabolism, immune regulation, and cytoskeletal organization, including *NAPRT*, *GRHL2*, *EHF*, *MET*, *OVOL2*, *SPINT1*, *ATP8B1*, *CD9*, *HMOX2*, and *TMPRSS2* (Figure 8e), jointly maintaining homeostasis of thyroid hormone synthesis and secretion and indirectly influencing reproductive performance through endocrine–metabolic pathways. Hub genes in the red module regulated uterine remodeling, blood supply, smooth muscle contraction, and embryo implantation, including *RSPO1*, *AGTR2*, *DKK2*, *ACTN1*, *ITGA1*, *PTGER3*, *CAV1*, *ENSA*, *HSPB6*, and *LMNA* (Figure 8f), cooperatively establishing uterine receptivity in HF goats and enhancing embryo implantation efficiency and pregnancy maintenance.

### 3.9. Integrative Summary of Transcriptomic and Metabolomic

Table 2 summarizes the key KEGG pathways, representative hub genes, and hub metabolites identified in the follicle, thyroid, and uterine horn. For each tissue, the DEG- and DEM-enriched pathways are listed alongside the WGCNA-derived hub genes and hub metabolites, enabling direct cross-omics comparison within the same tissue.

## 4. Discussion

In the present study, the larger litter size in HF goats, accompanied by a lower individual birth weight, reflects the inverse relationship between these traits which is consistent with intrauterine competition and resource partitioning reported previously [21,22]. Importantly, the birth weight disadvantage was compensated by rapid growth within the first month, indicating that early postnatal compensatory growth is sufficient to offset the intrauterine nutrient dilution effect associated with higher litter size, as has been observed in prolific breeds like the Hu sheep [23]. Our hormone profiling results showed that elevated E2 and FT3 levels during the follicular phase (day 0) in HF goats indicated enhanced steroidogenesis and HPT axis activity, supporting follicular development and oocyte maturation [24]. The concurrent elevation of P4, TT3, TT4, and P4/E2 ratio during the mid-luteal phase (day 10) in HF goats indicated enhanced corpus luteum function and improved follicular health, which are associated with successful embryo implantation and survival [24,25,26]. By contrast, LF goats showed a significant increase in TT3/TT4 ratio across most estrous stages (days 0, 4, and 16), suggesting a dysregulated negative feedback mechanism within the HPT axis. An elevated TT3/TT4 ratio has been associated with impaired T4 secretion or enhanced peripheral deiodinase activity, either of which can disrupt thyroid hormone homeostasis and the energy balance required for normal reproduction [27,28]. The concurrent TSH elevation further suggests dysregulated HPT axis feedback during the late luteal phase (day 16). Thyroid dysfunction, even at subclinical levels, has been linked to impaired folliculogenesis, anovulation, and reduced pregnancy rates in several species [29,30]. Our results show that HPT axis dysregulation in LF goats is temporally aligned with reproductive transitions, which may act as a systemic endocrine constraint on fecundity and offer direct evidence for thyroid–reproductive axis interactions. Uterine morphology analysis further demonstrated that LF goats were characterized by lower gland density, reduced myometrial thickness, and more ductal gland invaginations. Uterine glands serve as the primary source of histotroph supporting the developing conceptus before placentation, and the uterine gland density has been identified as a key determinant of uterine receptivity and embryo survival [31]. The higher DGI in LF goats may therefore reflect aberrant glandular morphogenesis or remodeling, potentially compromising the secretory capacity of the endometrium and reducing the probability of successful implantation.

We identified 6640 metabolites by non-targeted LC-MS metabolomics including amino acids, glycerophospholipids, organic acids, and hormone-related substances. DEMs exhibited tissue-specific distribution, with 271 metabolites in follicular fluid and only 83 in uterine luminal fluid. This pattern reflects the distinct metabolic environments and functional demands of each compartment, which is consistent with previous studies identifying follicular fluid as the most metabolically active reproductive biofluid [32,33]. KEGG pathway enrichment revealed that follicular fluid DEMs were primarily enriched in energy metabolism (TCA cycle, glycolysis/gluconeogenesis) and reproductive hormone biosynthesis (steroid hormone biosynthesis, neuroactive ligand–receptor interaction). These enrichment patterns reflect the well-established role of follicular fluid as a metabolic and energy substrate reservoir supporting oocyte maturation and granulosa cell function [34]. Serum DEMs were enriched in basal metabolic pathways (glycerophospholipid metabolism, cholesterol metabolism, pentose phosphate pathway), consistent with the role of systemic circulation as a global metabolic integrator. The thyroid exhibited the most pronounced and diverse pathway perturbations, including α-linolenic acid metabolism and, particularly, the co-enrichment of the PPAR signaling pathway (governing lipid metabolism and interacting with thyroid signaling [35,36]) and tyrosine metabolism (the direct precursor to thyroid hormones [37]). The simultaneous disruption of tyrosine metabolism and PPAR signaling indicates that defective lipid-endocrine coordination in the thyroid may contribute to the observed fecundity decline, supporting the thyroid as a vital endocrine and metabolic hub linking metabolism and reproductive function. Uterine luminal fluid DEMs were enriched in glycerophospholipid metabolism, histidine metabolism, and cysteine and methionine metabolism. Glycerophospholipids are critical for endometrial membrane remodeling and prostaglandin synthesis during the implantation window [38], while one-carbon metabolism (cysteine and methionine pathways) plays an important role in DNA methylation and epigenetic programming of the endometrium during the peri-implantation period [39,40]. These findings suggest that metabolic insufficiency in lipid remodeling and one-carbon metabolism may impair endometrial receptivity in LF goats at the local uterine level.

We identified 1596 DEGs across follicle, thyroid, and uterine horn by transcriptomic profiling, with 20 DEGs shared among all three tissues, constituting a systemic transcriptional signature of fecundity divergence. These genes could mediate critical reproductive processes: prostaglandin efflux and signaling attenuation (MRP4/ABCC4-like paralogs) [41], maternal immune tolerance (MHC class I, *TNFR*) [42,43], calcium-dependent neuroendocrine secretion (*CADPS*, *SYT4*) [44], extracellular matrix remodeling (*MMP3*, *NELL1*) [45], neuropeptide processing (*CPA6*) [46], and translational regulation (*RPS28*) [47]. We found that follicular DEGs were enriched in PI3K-Akt, calcium, ECM–receptor interaction, focal adhesion, and complement and coagulation cascade pathways, implicating these pathways in granulosa cell proliferation, follicle wall integrity, and local immune regulation during follicular maturation and ovulation [48,49,50,51,52]. The PI3K-Akt pathway is a well-established regulator of granulosa cell proliferation, follicle survival, and steroidogenesis [53]; calcium signaling modulates meiotic resumption in oocytes [54]; the cAMP pathway serves as the primary second messenger for FSH action in follicular cells [55]. In the present study, the concurrent enrichment of ECM-receptor interaction and focal adhesion pathways further underscores the importance of cell–matrix communication in follicular morphogenesis and ovulation [56]. In thyroid tissue, DEGs were enriched in autoimmune, steroid biosynthesis, and PPAR pathways, indicating subclinical immune dysregulation and disrupted thyroid hormone synthesis, which indirectly impairs reproductive function [57,58]. Autoimmune thyroid disorders are associated with anti-thyroid antibody production, which may disrupt thyroid hormone synthesis and secondarily affect GnRH pulsatility and gonadotropin secretion [12]. The steroidogenic enrichment observed here provides novel transcriptomic support for molecular interaction between the thyroid and reproductive axes, such as thyroid hormone-driven suppression of BMP-6 signaling to modulate granulosa cell steroidogenesis [59]. In the uterine horn, we found DEGs converging on cAMP signaling, calcium signaling, cytokine–cytokine receptor interaction, neuroactive ligand-receptor interaction, and complement/coagulation cascades, which collectively orchestrate the immunological, vascular, and contractile changes required for endometrial receptivity. The cGMP-PKG signaling pathway and platelet activation were specifically enriched in uterine tissue, consistent with their roles in regulating myometrial quiescence and blood flow during early pregnancy [60,61]. The endometrial receptivity transcriptome in goats has been characterized in several recent studies, which identified cAMP and calcium signaling as central pathways during the window of implantation [62,63,64].

We used WGCNA to identify tissue-specific co-expression modules in both metabolite and gene networks. Three metabolite modules (red, black, pink) and three gene modules (black, green, red) were prominently associated with LF or HF groups. The convergence of KEGG pathway enrichment between metabolite modules (neuroactive ligand-receptor interaction, nucleotide metabolism, PPAR signaling, arachidonic acid metabolism) and gene modules (ECM-receptor interaction, focal adhesion, cAMP signaling, steroid biosynthesis, oxytocin signaling) supports the functional coherence of the metabolite-gene regulatory co-network.

The follicular development axis is defined by the red metabolite module and black gene module, both positively correlated with LF follicles. At the metabolite level, polyunsaturated fatty acids (e.g., 13,16,19-docosatrienoic acid) provide lipid-metabolic substrates, while purine metabolites (e.g., hypoxanthine) may supply energy for follicular cell proliferation. Among the hub genes identified in this module, *ELOVL4* catalyzes very long-chain polyunsaturated fatty acids (VLC-PUFA) synthesis [65], linking the metabolite module to steroid hormone biosynthesis. *INHA* encodes a gonadal-derived glycoprotein that inhibits the production and release of follicle-stimulating hormone (FSH) [66]. *NR5A2* transcriptionally controls aromatase (*CYP19A1*) and estradiol synthesis [67]. *SCD5* regulates fatty acid desaturation, modulating membrane fluidity and steroidogenic capacity in granulosa cells [68]. Additional support for follicular homeostasis is achieved via luteal regulation (*OXT*), amino acid metabolism (*GPT*), pH balance (*SLC4A3*), iron uptake (*TFR2*), fatty acid oxidation (*ABCD1*), and meiotic synapsis (*SYCP1*). Notably, the black gene module’s correlation with LF rather than HF suggests that the follicular transcriptional program in low-fecundity goats reflects a compensatory or dysregulated state. The thyroid endocrine axis integrates the black metabolite module and green gene module, both positively correlated with LF thyroid. Succinate modulates thyroid energy metabolism via regulating mitochondrial protein succinylation [69], and *NAPRT* supports this process as the rate-limiting enzyme for NAD+ synthesis [70]. At the gene level, *GRHL2* may play a key role in epithelial integrity and glandular morphogenesis [71], and its expression has been confirmed in thyroid glandular cells. Beyond its structural role, *GRHL2* also acts as a transcriptional co-regulator, potentially influencing steroid receptor-mediated gene expression [72]. Additional hub genes are predicted to play roles in maintaining thyroid epithelial barrier integrity (*ENF*, *OVOL2*, *SPINT1*), sustaining membrane stability and hormone secretion (*CD9*, *ATP8B1*), regulating cellular metabolism and signaling (*MET*, *HMOX2*), and remodeling the extracellular microenvironment (*TMPRSS2*). This metabolite–gene convergence provides a molecular basis for endocrine–reproductive cross-regulation. The uterine receptivity axis is defined by the pink metabolite module (LF-associated) and the red gene module, which is uniquely correlated with the HF group. At the metabolite level, Prostaglandins promote decidualization and endometrial receptivity [73], and certain acylcarnitines are associated with metabolic changes in the receptive uterus [74]. Their enrichment in the pink module suggests that dysregulated prostaglandin signaling may compromise uterine receptivity in LF does. At the gene level, *RSPO1* activates Wnt/β-catenin signaling to maintain endometrial receptivity [75], and *AGTR2* mediates uterine arterial vasodilation to augment implantation-site blood supply [76]. *ACTN1*, *ITGA1*, *CAV1*, and *PTGER3* have been implicated in actin cytoskeletal dynamics, cell–matrix adhesion, and prostaglandin receptor signaling, and *PTGER3* is likely to mediate uterine contraction [77,78,79]. Complementary hub genes further consolidate this advantage: *DKK2* modulates Wnt-dependent processes crucial for embryo implantation [80], *HSPB6* promotes smooth muscle relaxation and vascular tone [81], and *LMNA* maintains nuclear integrity in endometrial stromal cells [82], collectively providing a molecular basis for superior uterine receptivity in HF goats. While low-fecundity signatures reflect systemic dysregulation centered on follicular and thyroid function, the high-fecundity phenotype is uniquely characterized by a uterine-specific transcriptional program oriented toward enhancing endometrial receptivity.

Although the sample size for transcriptomic analysis was relatively limited (*n* = 3 per group), which may reduce the sensitivity for detecting DEGs with moderate fold changes and affect the stability of WGCNA module assignments, stringent statistical thresholds, integrated multi-omics analysis, and network-based validation strategies were applied to enhanced the robustness and biological reliability of the findings. The present study identified associations between hub molecules and fecundity through correlative network analysis. Whether these molecules are causally involved in fecundity requires experimental validation. Therefore, future studies should: (1) employ expanded omics (e.g., lncRNA, epigenomics) to delineate upstream regulation; (2) functionally validate key hubs via genetic or pharmacological models; and (3) translate the identified gene-metabolite panels into biomarkers for early selection, and target the dysregulated pathways (e.g., PPAR signaling) for nutritional intervention.

## 5. Conclusions

This study provides the first integrated metabolomic and transcriptomic framework describing the molecular basis of differential fecundity in Yangtze River Delta White goats. Our findings suggest that fecundity involves coordinated interactions among follicular development, thyroid endocrine regulation, and uterine receptivity. Key hub genes, including *ELOVL4*, *GRHL2*, and *RSPO1*, together with metabolites such as hypoxanthine, succinic anhydride, and prostaglandin D2, are associated with steroidogenesis, epithelial integrity, endocrine homeostasis, and implantation-associated signaling pathways.

Mechanistically, low fecundity was characterized by impaired follicular development and instability of the HPT axis, whereas high fecundity was associated with enhanced uterine receptivity and improved implantation-supportive transcriptional activity. The identified genes and metabolites may serve as candidate biomarkers for fecundity assessment, pending validation in larger populations. Overall, this study advances current understanding of reproductive systems biology in goats and provides a molecular framework that may inform future studies on biomarker-assisted selection and targeted reproductive management.

## Figures and Tables

**Table 1:** Comparison of reproductive traits in goats with different fecundity.

Items	LF	HF	*p*-Value
Estrus duration (h)	38.18 ± 2.46	42.75 ± 1.36	0.628
Estrus cycle (d)	20.18 ± 0.35	19.97 ± 0.17	0.163
Gestation sacs (35 d)	1.63 ^a^ ± 0.18	3.14 ^b^ ± 0.42	0.004
Gestation sacs (50 d)	1.88 ^a^ ± 0.30	3.00 ^b^ ± 0.34	0.010
Gestation sacs (70 d)	2.14 ^a^ ± 0.14	3.00 ^b^ ± 0.32	0.021
Gestation period (d)	144.71 ^a^ ± 2.07	150.25 ^b^ ± 1.25	0.035
Litter sizes	1.67 ^a^ ± 0.24	3.00 ^b^ ± 0.24	0.001
Litter weight (kg)	3.42 ^a^ ± 0.36	4.87 ^b^ ± 0.30	0.001
Individual birth weight (kg)	2.16 ^a^ ± 0.14	1.67 ^b^ ± 0.12	0.015
Litter weight at 1 month (kg)	5.02 ± 0.60	6.76 ± 1.55	0.342
Individual weight at 1 month (kg)	4.22 ± 0.16	4.30 ± 0.17	0.724
Weaning litter weight (kg)	7.59 ± 1.12	9.46 ± 1.55	0.336
Individual weaning weight (kg)	6.60 ± 0.52	6.45 ± 0.65	0.867

Note: Data are presented as mean ± standard error of the mean (SEM). Within each row, different superscript letters (a, b) indicate significant differences (*p* < 0.05).

**Table 2:** Summary of key tissues, pathways, hub genes, and hub metabolites associated with differential fecundity.

Tissue	Key KEGG Pathways of DEGs	Hub Genes	Key KEGG Pathways of DEMs	Hub Metabolites
Follicle	PI3K-Akt, Calcium signaling, cAMP, Neuroactive ligand–receptor interaction, ECM–receptor interaction, Focal adhesion, Complement and coagulation cascades.	*ELOVL4*, *SCD5*, *INHA*, *OXT*, *GPT*	TCA cycle, Steroid hormone biosynthesis, Neuroactive ligand–receptor interaction, Amino sugar and nucleotide sugar metabolism, Glucagon signaling pathway, Biosynthesis of nucleotide sugars.	Hypoxanthine, 5′-GMP, Phytanic acid, Estradiol DGLA-EA.
Thyroid	Autoimmune thyroid disease, Ovarian steroidogenesis, Steroid hormone biosynthesis, cAMP, Neuroactive ligand–receptor interaction, PPAR signaling, Retinol metabolism, Arachidonic acid metabolism.	*NAPRT*, *GRHL2*, *EHF*, *MET*, *OVOL2*	PPAR signaling pathway, Tyrosine metabolism, α-Linolenic acid metabolism, Glycerophospholipid metabolism, Linoleic acid metabolism, Metabolic pathways.	Succinic anhydride, β-Glycerophosphoric acid, Oxobutanoic acid, 5′-UMP, Polymannose.
Uterine Horn	cAMP, Calcium signaling, Neuroactive ligand–receptor interaction, cGMP-PKG, Complement and coagulation cascades, Platelet activation, Focal adhesion	*RSPO1*, *AGTR2*, *DKK2*, *ACTN1*, *PTGER3*	Glycerophospholipid metabolism, GPI-anchor biosynthesis, Histidine metabolism, Cysteine and methionine metabolism, Arachidonic acid metabolism, Autophagy.	Prostaglandin D2, Prostaglandin F3α, 11β-Prostaglandin F2α, N6-Succinyl Adenosine. LTB4.

Note: DGLA-EA: Dihomo-gamma-linolenoylethanolamide.

## Data Availability

The raw sequence data reported in this paper have been deposited in the Genome Sequence Archive (Genomics, Proteomics & Bioinformatics 2025) in National Genomics Data Center (Nucleic Acids Res 2025), China National Center for Bioinformation/Beijing Institute of Genomics, Chinese Academy of Sciences (GSA: CRA041756) that are publicly accessible at https://ngdc.cncb.ac.cn/gsa, accessed on 28 June 2026.

## Supplementary Materials

The following supporting information can be downloaded at: https://www.mdpi.com/article/10.3390/ani16132034/s1, Figure S1: OPLS-DA permutation test plot. Figure S2: Determination of the soft-thresholding power of metabolite co-expression modules. (a) Scale-free topology fit index (R^2^); (b) Mean connectivity. Figure S3: Determination of the soft-thresholding power of gene co-expression modules. (a) Scale-free topology fit index (R^2^); (b) Mean connectivity. Table S1: The primers used for RT-qPCR. Table S2: Identification of total metabolites in four tissues of goats. Table S3: Identification of total metabolites in LFF vs. HFF. Table S4: Identification of total metabolites in LFS vs. HFS. Table S5: Identification of total metabolites in LFT vs. HFT. Table S6: Identification of total metabolites in LFUL vs. HFUL. Table S7: Metabolomic KEGG functional enrichment analysis in LFF vs. HFF. Table S8: Metabolomic KEGG functional enrichment analysis in LFS vs. HFS. Table S9: Metabolomic KEGG functional enrichment analysis in LFT vs. HFT. Table S10: Metabolomic KEGG functional enrichment analysis in LFUL vs. HFUL. Table S11: Expression profiles of 29,327 genes detected in three tissues of goats. Table S12: Overview of DEGs detected in LFF vs. HFF. Table S13: Overview of DEGs detected in LFT vs. HFT. Table S14: Overview of DEGs detected in LFZ vs. HFZ. Table S15: 20 DEGs common to three tissues of goats; Table S16: Hub metabolites; Table S17: Hub genes.
