# Supplementary material for: Integrative Transcriptomic and Metabolomic Analysis Reveal Mechanisms Underlying Differential Fecundity in Yangtze River Delta White Goat
Source: Animals (Basel). 2026 Jul 2;16(13):2034. doi: 10.3390/ani16132034 (PMC13359923; doi:10.3390/ani16132034)
Supplement: Supplementary file 1 [file animals-16-02034-s001.zip › Supplementary Figure S2.Determination of the soft‐thresholding power of metabolite co‐expression modules. (a)Scale‐free topology fit index (R2); (b) Mean connectivity.pdf]

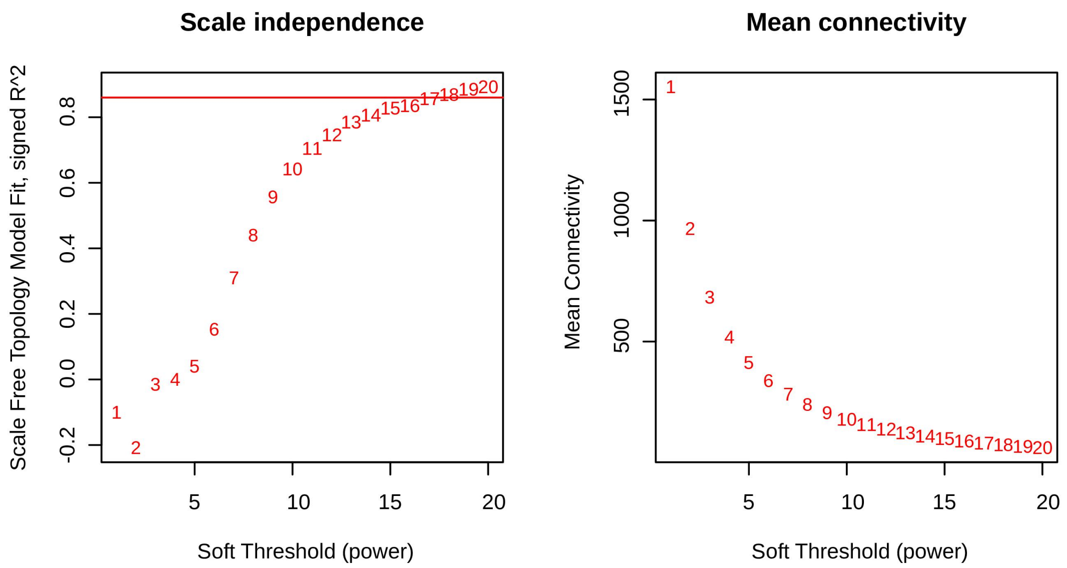

Figure S2. Determination of the soft-thresholding power of metabolite co-expression modules. (a) Scale-free topology fit index (R^2); (b) Mean connectivity.
