# Supplementary figures and images for: Integrative Transcriptomic and Metabolomic Analysis Reveal Mechanisms Underlying Differential Fecundity in Yangtze River Delta White Goat
Source: Animals (Basel). 2026 Jul 2;16(13):2034. doi: 10.3390/ani16132034 (PMC13359923; doi:10.3390/ani16132034)

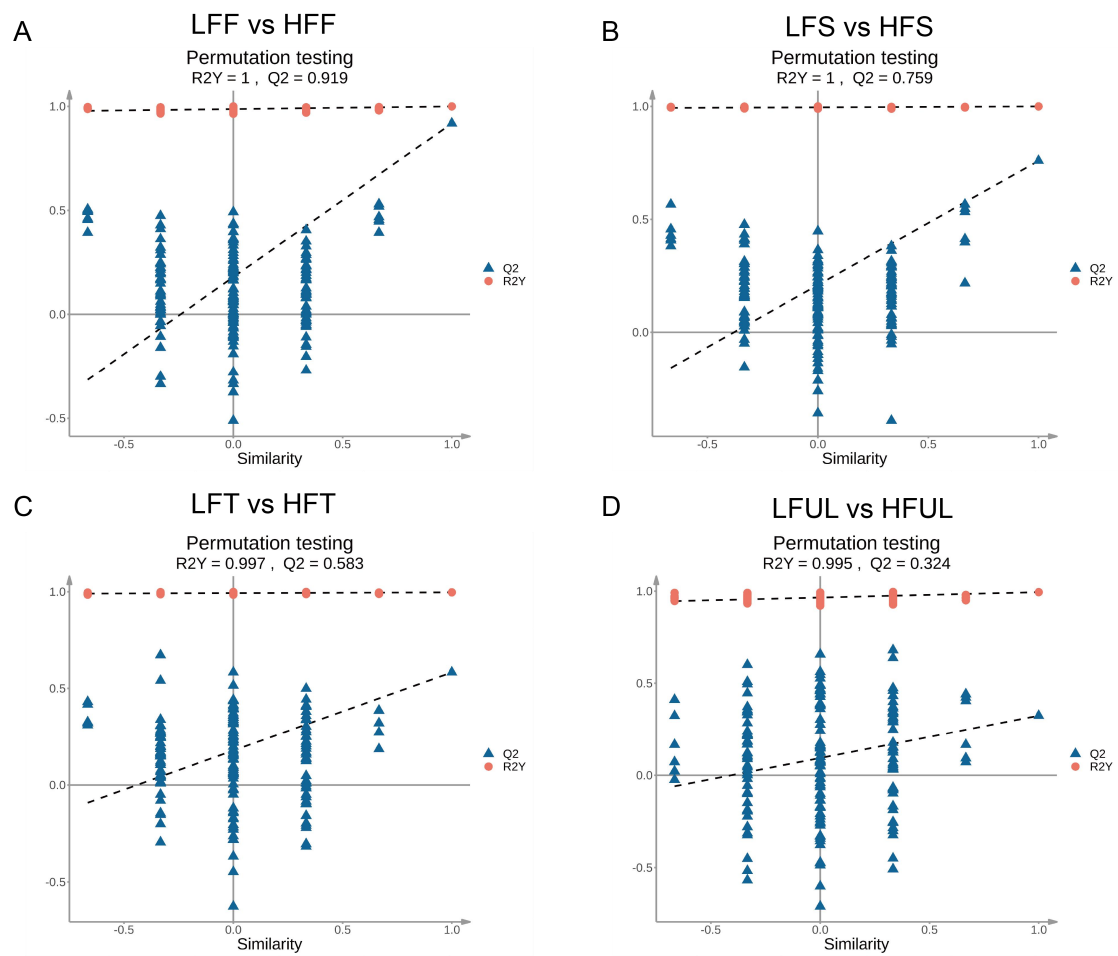

Figure S1. OPLS-DA permutation test plot

Supplement: Supplementary file 1 [file animals-16-02034-s001.zip › Supplementary Figure S1.Supplementary Figure S1.OPLS-DA permutation test plotpdf.pdf]
